# Surviving Pneumonia: A Rare Outcome of Compensatory Hyperinflation in Fibrotic Lung Disease

**DOI:** 10.7759/cureus.39771

**Published:** 2023-05-31

**Authors:** Mukesh Kumar, Prateek K Dinkar, Vinay Suresh, Shiva Gupta, Vaibhav Singh, Haider Abbas, Esha Chaudhary, Pranay Gupta

**Affiliations:** 1 Emergency Medicine, King George's Medical University, Lucknow, IND; 2 General Medicine, King George's Medical University, Lucknow, IND; 3 Faculty of Medical Sciences, King George's Medical University, Lucknow, IND; 4 Faculty of Medicine, Government Medical College, Kannauj, Kannauj, IND

**Keywords:** radiculopathy, lower limb pain, pneumonia, fibrosis, compensatory hyperinflation

## Abstract

Chronic respiratory insufficiency can result from respiratory infections like pneumonia, which can permanently harm the lungs and respiratory system. A 21-year-old female patient arrived at our emergency medicine department (ED) complaining of acute lower-limb pain that worsened when she walked. She also reported feeling weak and having an acute, undiagnosed fever that was resolved by taking medicine two days after the day of admission. She was found to have a body temperature of 99.4°F, decreased air entry on the left side of the chest, and diminished bilateral plantar responsiveness. With the exception of a low calcium level and an increased liver function test, her biochemical indicators were normal.

The left lung's basal region had fibrosis, and the right lung's hyperplasia served as a compensatory mechanism, according to the chest radiograph and CT scan of the thorax. The patient underwent treatment with intravenous pantoprazole, ondansetron, ceftriaxone, multivitamin supplementation, gabapentin, and tablets of amitriptyline. On Day 7, her lower limb pain had significantly recovered. After an eight-day hospital stay, she was discharged with instructions to follow up with the pulmonary medicine outpatient department (OPD) and the neurology OPD. A well-known occurrence known as compensatory hyperinflation of the lung happens when one lung is severely injured or rendered inoperable, leading the other lung to enlarge to make up for the loss of respiratory function. This case demonstrates the ability of the respiratory system to compensate for significant damage to one of the lungs.

## Introduction

Pulmonary hyperinflation is defined as an abnormal rise in functional residual capacity (FRC) of the lung, which is the volume of the lung at the end of tidal expiration. To a radiologist, however, hyperinflation implies an increase in total lung capacity. Several conditions that cause significant unilateral destruction of the lung can also result in significant compensatory hyperinflation on the other side. When a lung is severely hyperinflated, it frequently herniates anteriorly across the midline and may almost completely fill the opposite hemithorax. This phenomenon is referred to as pseudo-horseshoe lung [1]. Horseshoe lung, on the other hand, is a rare congenital defect characterized by a midline pulmonary parenchyma isthmus that connects the lungs. Although pseudo-horseshoe lung typically affects children, adults can also be diagnosed with asymptomatic cases of the condition [2]. In this case report, we presented a 21-year-old female patient, who was referred to us with a chief complaint of pain in the bilateral lower limb. The patient had suffered from severe pneumonia in the past. Following that, it was discovered that she had severe pneumonia-induced fibrosis on the left side, accompanied by compensatory hyperinflation of the right lung. A well-known occurrence known as compensatory hyperinflation of the lung happens when one lung is severely injured or rendered inoperable, leading the other lung to enlarge to make up for the loss of respiratory function. This case shows the potential for the lungs' compensatory power to recover fully even after considerable damage has occurred in one of the lungs.

## Case presentation

A 21-year-old female presented to the emergency medicine department (ED) with complaints of pain in bilateral lower limbs for one month, acute in onset, progressive in nature, aggravated on walking, non-relieving, and associated with generalized body weakness. Furthermore, she added an undocumented fever, acute in onset, on and off in nature, without diurnal variation, chills, or rigors, and relieved on taking acetaminophen 500 mg twice a day, two days prior to the day of admission. There was no history of trauma. Upon further review of history, it was found that the patient had suffered from severe pneumonia at the age of three years. The patient had no respiratory discomfort. The patient had no history of diabetes, hypertension, tuberculosis, asthma, thyroid disorder, or significant surgical intervention if any. There was no history of allergy or contact with tuberculosis patient.

On physical examination, she looked unwell, with a physical temperature of 99.4°F, heart rate of 98 beats per minute, blood pressure of 119/79 mmHg, and oxygen saturation of 98% on room air. On respiratory examination, air entry was reduced on the left side of the chest at the basal region. On cardiovascular examination, normal S1 and S2 sounds with no added sounds or murmur were present. The central nervous system examination revealed a grade 1 sensorium with a Glasgow Coma Score (GCS) of 15. Both pupils were reactive to light. She was moving her limbs on command but could not walk due to pain in her bilateral lower limbs. Her deep tendon reflexes were intact but bilateral plantar response was reduced. Detailed motor and sensory system examinations were carried out and found to be intact. There was no neck stiffness. We closely kept monitoring related parameters until our patient was discharged.

On Day 1 of admission, her biochemical markers were normal except for the liver function test, where serum glutamic oxaloacetic transaminase (SGOT) was 64.9 IU/L (0-40 IU/L) and serum glutamic pyruvic transaminase (SGPT) was 54.5 IU/L (0-45 IU/L). The serum electrolyte showed a reduced calcium level of 3.56 mg/dl (4.5-5.5 mg/dl). The serum vitamin B12 level was 2134 pg/ml (Normal: 187-883 pg/ml; Deficiency: 32-187 pg/ml).

**Figure 1:**
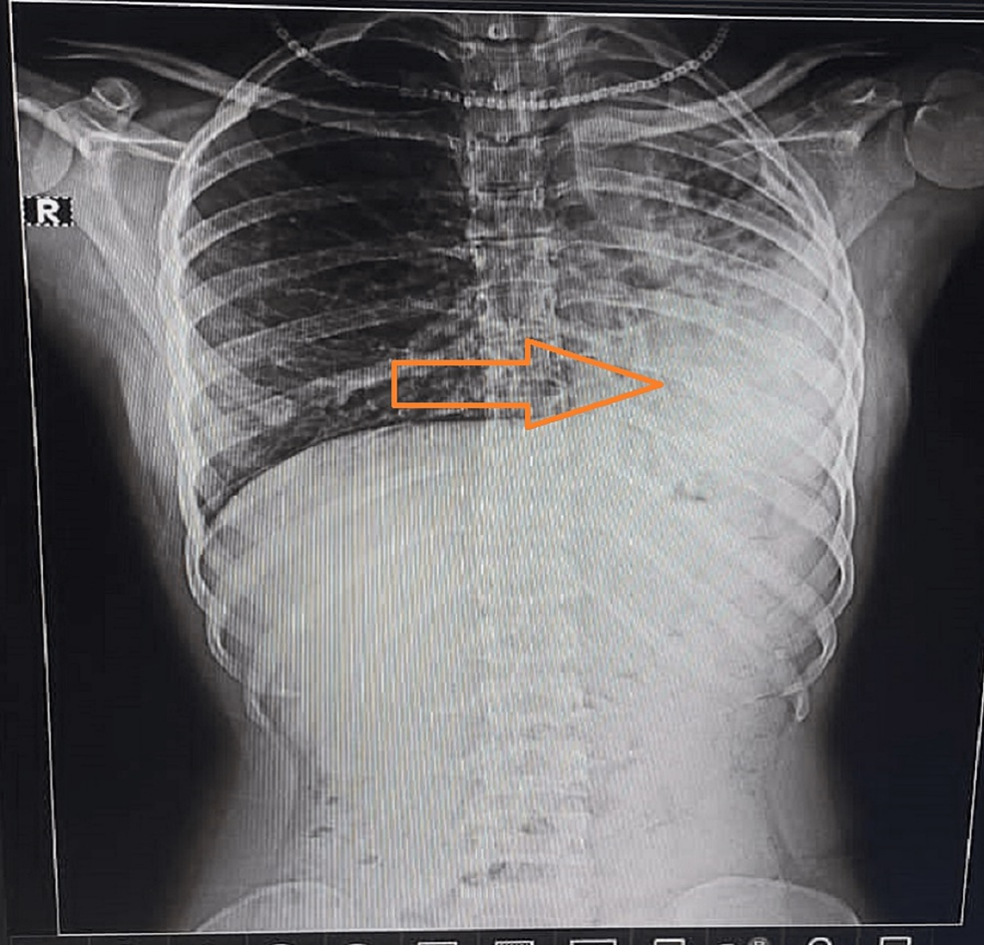
PA view chest X-ray showing left-sided basal lung fibrosis PA: posteroanterior

**Figure 2:**
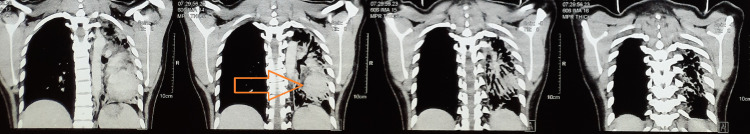
HRCT showing left-sided basal lung fibrosis HRCT: high-resolution computed tomography

The chest radiograph indicated the presence of fibrosis in the basal area of the left lung, as depicted in Figure 1. To aid in establishing the diagnosis, including high-resolution computed tomography (HRCT) images showing hyperinflation in the right lung is recommended. Additionally, during further evaluation using a CT scan of the thorax, evidence of volume loss on the left side and compensatory hyperinflation of the right lung was observed while normal lung tissue was identified in the respective region (Figure 2). These observations were consistent with a diagnosis of left lung fibrosis, believed to have arisen as a sequel to severe pneumonia.

Treatment consisted of intravenous (IV) pantoprazole 40 mg BD, IV ondansetron 4 mg BD, IV tramadol 1 ampule in 100 ml normal saline (NS) stat, multivitamin supplementation, and IV ceftriaxone 1 gm BD, on Day 1 of admission. On Day 2, we revoked tramadol, as she was found allergic to it. Her color Doppler test was normal and without any evidence of deep venous thrombosis (DVT).

**Figure 3:**
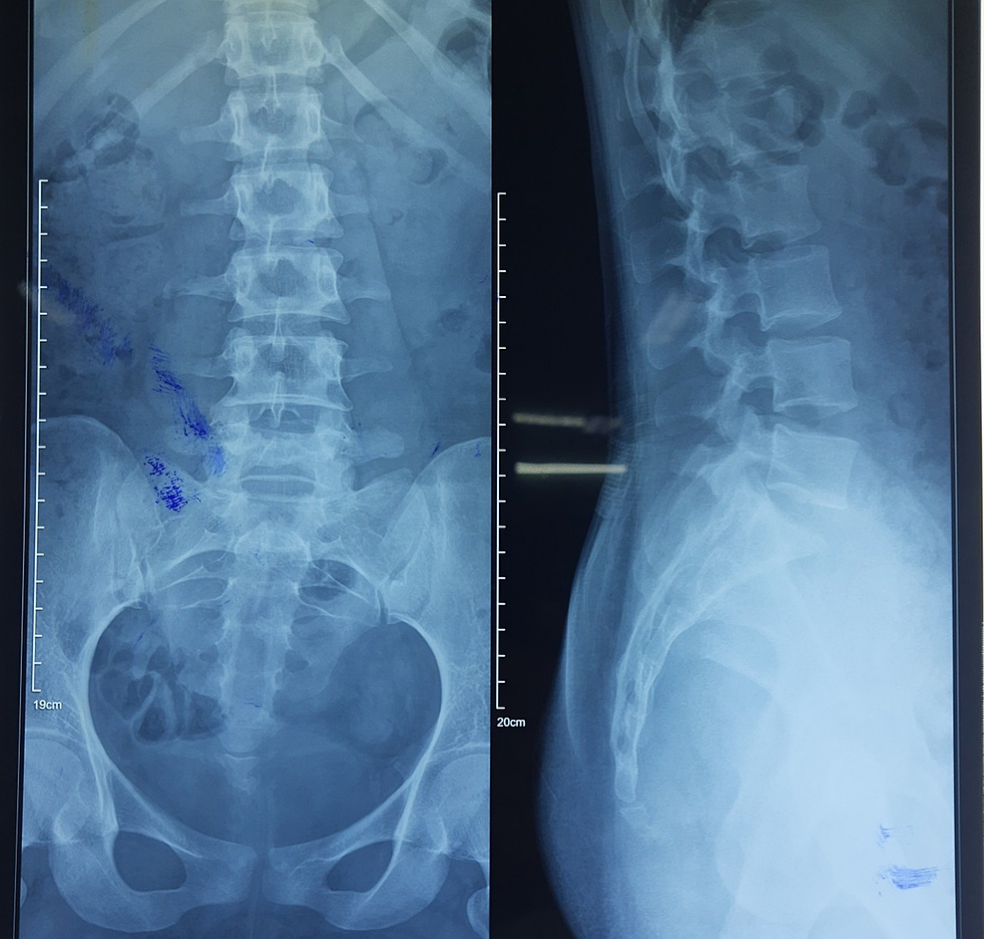
X-ray of the lumbosacral spine (LS) from the AP view (left) and lateral view (right) AP: anteroposterior

On Day 3, the patient underwent a neurology consultation, during which compressive radiculopathy was suspected. As a result, gabapentin tablets of 100 mg strength were added to the patient's regimen to be taken once daily at bedtime, along with amitriptyline tablets of 10 mg strength, also to be taken once daily at bedtime. An X-ray of the lumbosacral (LS) spine (in anteroposterior (AP) and lateral views) was subsequently conducted for further assessment and was found normal (Figure 3). On Day 4, she was hematologically stable and her lower limb pain also started to improve. On Day 5, she could manage to walk under the observation of a physiotherapist. On Days 6 and 7, her hematological parameters were normal. She was maintaining a 98% oxygen saturation level on room air. She was having a mild degree of pain while walking. She was able to walk to the washroom on her own. She was discharged after an eight-day stay in the hospital with advice to follow up in the respiratory medicine outpatient department (OPD) and neurology OPD.

## Discussion

Compensatory hyperinflation of the lung is a well-known phenomenon that occurs when one lung is severely damaged or non-functional, causing the other lung to expand to compensate for the loss of respiratory function [3]. This can be caused by various conditions such as bronchiectasis, cystic fibrosis, and emphysema [4]. When a lung is severely hyperinflated, it may herniate anteriorly across the midline, leading to a horseshoe-shaped appearance on chest radiographs, which is referred to as a pseudo-horseshoe lung [5]. On the other hand, a horseshoe lung is a rare congenital defect characterized by a midline pulmonary parenchyma isthmus that connects the lungs [6]. Although a pseudo-horseshoe lung typically affects children, asymptomatic cases of the condition can also be detected in adults.

Our patient presented with pain in the bilateral lower limbs and was found to have a left-sided pleural effusion with fibrosis and compensatory hyperinflation of the right lung. The patient had a history of past pneumonia, but there was no other significant medical or surgical history. The chest X-ray and CT scan of the thorax demonstrated significant hyperinflation of the right lung, which could be the result of the compensatory mechanism due to damage to the left lung [6].

The differential diagnosis for hyperinflation of the lung includes congenital disorders such as horseshoe lung and bronchopulmonary dysplasia, chronic obstructive pulmonary disease (COPD), asthma, and cystic fibrosis. In this case, there was no evidence of any congenital defects or any chronic pulmonary diseases, and the patient had no history of asthma or cystic fibrosis. Furthermore, there was no evidence of any acute exacerbation of COPD [7].

The patient was diagnosed with left-sided pleural effusion with fibrosis, which could be the result of a previous infection or inflammation. Fibrosis can develop as a result of prolonged inflammation in the pleural space, which can lead to decreased lung compliance and restrict the lung's ability to expand, thereby resulting in compensatory hyperinflation of the contralateral lung.

## Conclusions

Compensatory hyperinflation is a physiological phenomenon where a functional lung expands to compensate for the loss of respiratory function in a damaged or non-functional lung. In this case, the patient had left-sided pleural effusion and fibrosis due to pneumonia when they were three years old. The fibrosis had affected the left lung, rendering it non-functional, but the right lung underwent compensatory hyperinflation to maintain adequate respiratory function. Despite the severity of the fibrosis, the patient's right lung exhibited an impressive compensatory capacity, resulting in complete functional recovery.

This case highlights the adaptability and compensatory ability of the lungs, even after a history of pneumonia and lung fibrosis. Understanding this compensatory ability can be useful in managing and treating lung diseases, as it may inform therapeutic strategies aimed at promoting the growth and development of lung tissue to enhance respiratory function. Although the patient presented with complaints related to a neurological disorder, a significant respiratory disorder was diagnosed. While there is limited information on the relationship between these two conditions, this case report could provide valuable insights and contribute to further understanding.

## References

[1] Madan K, Singh N (2013). Massive compensatory hyperinflation. BMJ Case Rep.

[2] Singh N, Agarwal R, Gupta D (2008). Pseudohorseshoe lung. CMAJ.

[3] Martinez FJ Lung Volume Reduction Surgery in COPD. UpToDate.

[4] Gagnon P, Guenette JA, Langer D (2014). Pathogenesis of hyperinflation in chronic obstructive pulmonary disease. Int J Chron Obstruct Pulmon Dis.

[5] Thuong Vu L, Duc NM, Tra My TT, Tuan Linh L, Quynh Huong T, Tan Lien Bang M (2021). Two rare cases of horseshoe lung with scimitar syndrome in Vietnam. Respir Med Case Rep.

[6] (2023). Spinal cord trauma. https://medlineplus.gov/ency/article/001066.htm.

[7] Gonen KA, Canitez Y, Bostan OM, Yazici Z (2019). Horseshoe lung associated with scimitar syndrome. BMJ Case Rep.

